# Characteristics and prediction of risky gambling behaviour study: A study protocol

**DOI:** 10.1002/mpr.1995

**Published:** 2023-11-04

**Authors:** Robert Czernecka, Theresa Wirkus, Gerhard Bühringer, Anja Kräplin

**Affiliations:** ^1^ Technische Universität (TU) Dresden Dresden Germany

**Keywords:** aggregated player tracking data, gambling disorder, longitudinal design, risk factors

## Abstract

**Objective:**

This study protocol describes the RIGAB study, a prospective case‐control‐study assessing online sports betting behaviour and underlying risk factors for the development of gambling disorder (GD). It has two aims: (1) to characterise sports bettors concerning putative risk factors and their gambling behaviour, and (2) to predict the development of GD from these factors.

**Methods:**

At baseline, online sports bettors took part in an online survey comprising a GD screening (DSM‐5), questions on gambling behaviour and on the putative risk factors emotion regulation, impulsivity, comorbidities, stress, and substance use. Participants were reinvited for a 1‐year follow‐up online survey. In a nested design, a subsample was invited in‐person to take part in a cognitive‐behavioural task battery and a clinical interview.

**Results:**

Of the initial 6568 online sports bettors invited, 607 participated at baseline (rate: 9.2%), 325 took part in the 1‐year follow‐up and 54 participated in the nested in‐person assessment.

**Conclusion:**

The RIGAB study combines different fields of GD studies: player tracking data and putative risk factors from self‐report and behavioural tasks. The results of this study will support the development of preventive measures for participants of online gambling based on the combined findings from previously rather distinct research fields.

## 1 INTRODUCTION

This study protocol describes the background, methods, and potential implications of the first longitudinal study on risk factors for gambling disorder (GD) in online sports bettors in Germany (RIGAB study). This topic is of growing importance as online gambling is an ever‐growing market within the EU gambling market with sports betting as one of the most popular products (European Gaming and Betting Association (EGBA), 2022). It is always accessible and, depending on the European country, either legal, or legal for some products or illegal (EGBA, 2022). In Germany, it has been fully legalised in all 16 federal states in the summer of 2021 (GlüStV, 2021). One of the reasons for legalisation was ‐as in other countries‐ the aim to better regulate online gambling and therefor to better prevent the development of GD. According to Auer and Griffiths (2022), Europe has some of the strictest player protection regulations. At the same time, there is still a lot of room for the improvement of prevention strategies within these regulations, that is, to base the early identification of GD more scientifically on the aetiology of GD. The RIGAB study wants to contribute to this improvement by adding knowledge on the risk profiles of online sports bettors concerning GD. In this section, we provide an overview on the definitions of GD and risky gambling behaviour in our study and on the research needs that will be addressed with the study.

### 1.1 Definitions: GD and risky gambling behaviour

We planned to focus our study onto those players who report clinically significant behaviour based on the current criteria for GD from the diagnostic and statistical manual for mental disorders (DSM‐5; American Psychiatric Association (APA), 2013). These behaviours include losing control over the temporal and financial dimensions of gambling, cognitive preoccupation with gambling and the neglect of important aspects of everyday life due to gambling (definition taken from our preregistered project overview, https://osf.io/b97ha).

Within the field of gambling studies, several terms (e.g., ‘problem gambling’, ‘moderate‐risk gambling’) are employed to describe gambling behaviour that results in adverse consequences for the individual or its environment (Neal et al., 2005). Apart from GD, these terms do not specify the clinical significance of the behaviour. Within the public health framework, these adverse consequences are often referred to as ‘harm’. The implied gambling behaviour is frequently conceptualised as being on a risk continuum, comprising behaviour below any clinical threshold as well as clinically significant behaviour (description taken from the preregistered initial online survey, https://osf.io/jbfhe). To conceptualise a lower bound threshold in this study, we defined risky gambling behaviour as any behaviour within gambling sessions that increases the probability of the development of GD or of voluntary self‐exclusion from gambling.

### 1.2 Research need 1: Characteristics of online sports bettors

The first research need we address is a much more holistic understanding of people who participate in online sports betting. So far, studies on player tracking data dominate the research field of online gambling. Studies on player tracking data could show that players with risky gambling behaviour ‐in comparison to controls‐ exhibit distinct gambling behaviour such as more frequent betting, a higher variability across the amounts wagered (Braverman & Shaffer, 2010), and placing a higher total number of bets (Gray et al., 2012). Several studies (for a systematic review see Deng et al., 2019) have been conducted mainly to find specific behavioural markers from player tracking data that correlate with markers for risky gambling behaviour, for example, number of different games played and subsequent account closure. All these studies have analysed player tracking data provided by online gambling platforms, some analysing bet by bet behaviour, others daily aggregates, some using machine learning for their analyses. However, this does not allow for conclusions about interindividual characteristics of the players, gambling motives, or whether online gamblers with certain characteristics fulfil a GD diagnosis.

So far, few studies have systematically assessed clinical and cognitive characteristics or individual risk factors of online gambling participants, that is, through clinical screenings, questionnaires, interviews, or lab experiments and have associated these findings to player tracking data. An exception is an Australian study (Russell, Hing, & Browne, 2019; Russell, Hing, Li, et al., 2019) using a big panel sample that compared Problem Gambling Severity Index (PGSI) screened sports bettors (online and offline) regarding a wide range of gambling behaviours ‐not including player tracking data‐ and a range of possible risk factors. In a more recent development, more studies use clinical screenings to compare players with and without GD. Louderback et al. (2021) for example, used a sample from 2010 that had filled in the Brief Biosocial Gambling Screen (BBGS; Gebauer et al., 2010) to develop lower risk online gambling thresholds. Luquiens et al. (2016) used player tracking data from poker players screened with the PGSI (Ferris & Wynne, 2001) developing an instrument that only uses player tracking data to predict problem gambling. Auer and Griffiths (2022) as well as Perrot et al. (2022) have also connected player tracking data to PGSI‐screened samples of online casino players to each develop algorithms that assess problem gamblers by their tracked online gambling behaviour.

In sum, we know about the players online gambling behaviour, sometimes in connection with clinical screenings. What is still needed is a better characterisation. We do not yet know a lot about online gamblers from a clinical point of view and how it all ties in with player tracking data and GD, which is an important research need to better target prevention and intervention measures to the needs of this group.

### 1.3 Research need 2: Prediction of GD in online sports betting

As reviewed in the previous section, player tracking data in online gambling are analysed to find risk factors in gambling behaviour as early behavioural markers of GD. Another line of research is aetiological research, which focuses on putative risk factors for the development of GD, that is, even before risky gambling behaviour has developed. Studies found various of these factors including altered emotion regulation (Jara‐Rizzo et al., 2019), various comorbidities such as substance use, affective and anxiety disorders (Dowling et al., 2015) and impulsivity (Chowdhury et al., 2017; Grant & Chamberlain, 2014; Weinsztok et al., 2021) across different forms of gambling. In a cross sectional study, Russell, Hing, and Browne (2019) found various putative risk factors specific to online and offline sports betting. Understanding the risk factors for GD in sports betting is an important area of research given the exponential growth of sports betting in many countries and the related public health concerns.

Regarding aetiological research in online gambling there is, on the one hand, research on player tracking data showing that certain aspects of gambling behaviour may predict GD. However, most of the studies were retrospective and in most of the studies GD was not the outcome; instead, proxies like net losses, total number of bet size and average duration of sessions were used (Deng et al., 2019). On the other hand, there are also many aetiological studies on putative risk factors of GD. These studies were however not specifically conducted in (online) sports betting and often cross‐sectional. Thus, there is a strong research need to analyse player tracking data on gambling behaviour and risk factors for GD in online sports bettors applying a prospective design. The RIGAB study is an opportunity to breach the gaps between the role of inter‐individual risk factors and player tracking data in GD and to contribute to the understanding of their causal role in the development of GD.

### 1.4 Research questions

In line with the aforementioned research needs, this study addresses the following two main research questions:

1) How are online sports bettors with and without GD characterised concerning player tracking data, putative individual, clinical and cognitive‐behavioural risk factors for GD, and behavioural markers for risky gambling behaviour?
2) Do these risk factors predict the development, that is, onset, progression, or remission of GD or of GD symptoms?

There are also four secondary research questions whose background we each briefly explain below:

1) Is our sample of online sports bettors with and without GD similarly characterised in terms of gambling behaviour and putative risk factors compared to previous studies?
  It is important to find out whether online gamblers differ from other gamblers in terms of risk factors as this would need to be considered when formulating prevention measures or therapy guidelines.
2) How stable is a GD diagnosis over time?
  A systematic review (Bischof et al., 2020) has shown that GD diagnosis is fairly unstable. It is important for us to gain more knowledge on the stability of GD specifically in online sports betting.
3) Are putative risk factors related to player tracking data of online sports bettors?
  Studies on player tracking data have shown that risky gambling behaviour is an early marker of GD (Braverman & Shaffer, 2010; Gray et al., 2012). There is a need to study the role of specific risk factors (such as emotion regulation or impulsivity) for GD in risky gambling behaviour as an early marker of GD. This would contribute to the understanding of their causal role in the development of GD. This research question is rather explorative, as an argument could be made that risk factors for GD, that is, impulsivity may strongly correlate with some betting behaviours (e.g., betting with riskier odds). It could also be that no clear pattern emerges, so that a lot of different risk factors are involved to predict gambling behaviour and there is no evidence for a strong relation.
4) What are the diagnostic properties of the artificial intelligence (AI) algorithm, the screening questionnaires, and the clinical interview regarding GD?
  Previous studies found large differences between screening instruments and diagnostic interviews concerning the prevalence of GD in casino gamblers (Kotter et al., 2019). To our best knowledge, there are no comparable studies on online sports bettors available. The same applies to studies reporting the validity of AI‐flagging concerning the detection of individuals with GD. To address this research need, we will compare the results of different screening instruments for GD used within the online surveys and the results of the clinical interview for GD to validate diagnostic instruments of GD. We will also be able to compare the provider's classification of players with and without risky gambling behaviour using AI to our classification of players with and without GD according to DSM‐5 criteria.

Based on the results concerning these questions, we will be able to inform etiological models of GD and probably enhance already existing preventive measures for GD. This will support the development of preventive measures for online sports betting and could be transferred to other kinds of online gambling.

## 2 METHODS AND MATERIALS

### 2.1 Design

**FIGURE 1:**
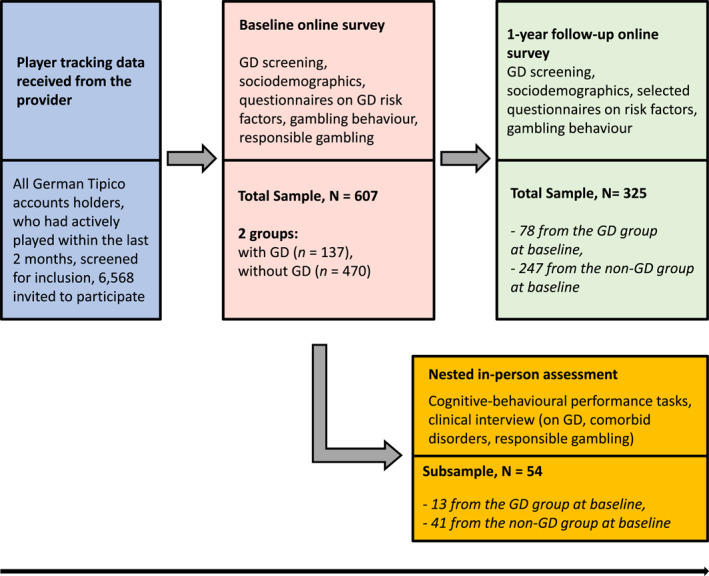
Study design of the RIGAB study (Czernecka et al., 2023b). GD = gambling disorder assessed with DSM‐5 Stinchfield screening questionnaire. RIGAB, risky gambling behaviour.

The RIGAB study is a prospective case‐control‐study with three parts, which have been preregistered: (1) an initial online survey (https://osf.io/jbfhe), (2) a follow‐up online survey (https://osf.io/k6c23/), and (3) a nested in‐person assessment consisting of a subsample (https://osf.io/g3nfv). The duration between baseline and follow‐up online survey was 1 year. The nested study part was conducted in‐person comprising a clinical interview and cognitive‐behavioural tasks. For the study design, see Figure 1 (Czernecka et al., 2023b). All participants were asked to provide informed consent at the initial online survey, which included later participation. The initial and the follow‐up online survey were conducted via the secure, web‐based software platform Research Electronic Data Capture (REDCap; Harris et al., 2009, 2019), which is hosted on a server of the Technische Universität Dresden. For the in‐person assessment, interviews and cognitive‐behavioural tasks were conducted in our lab in Dresden and in a collaborative lab in Berlin. At the end of each survey, participants had the opportunity to choose whether they wanted to have standardised feedback on their GD screening (For more details, see preregistration: https://osf.io/jbfhe). All participants chose whether they wanted to receive a voucher as remuneration for participation.

The Institutional Review Board (IRB00001473) of TU Dresden has approved the study protocol under the reference SR‐EK‐190032021.

### 2.2 Recruitment and sampling

Target sample sizes were 300 cases and 300 controls for the baseline initial online survey, 100 cases and 100 controls for the follow‐up online survey, and 40 cases and 40 controls for the nested in‐person assessment (for the sample size calculation, see preregistration: https://osf.io/jbfhe). We cooperated with Tipico Co. Ltd., an international provider of online sports betting, to recruit our participants. To select a random sample of online sports bettors, we received the player tracking data of all eligible customers from Tipico before the start of the initial baseline online survey. Tipico had provided us with anonymised data from all German online sports betting account holders who had logged into their accounts in the 2 months prior to the beginning of the study. Account data provided by Tipico included amongst others age, registration date, and place of residence.

Having obtained the player tracking data, we preselected account holders that met the following inclusion criteria: online sports bettors aged between 18 and 55 years and living close to one of the study locations in Germany. The potential participants were limited to six major German cities (Berlin, Chemnitz, Dresden, Hamburg, Leipzig, Munich) so that conducting the nested in‐person assessment could be more easily facilitated. We have no knowledge of significant differences amongst online sports betting account holders pertaining to different regions in Germany. Different to the preregistration, we later also preselected cases from two additional cities (Dusseldorf, Frankfurt), because not enough AI‐flagged account holders (see next paragraph) had been participating in the survey, yet. As a last inclusion criterion, the current account with the provider had to have a minimum age of 6 months. After preselection, we randomly chose account holders as participants for our study. For data protection reasons, the provider invited the participants to take part in the initial online study.

We planned to compare online sports bettors with and without GD. The prevalence for gamblers with GD at the time sampling was conducted had been reported with 0.5% for the last 10 years in Germany (Banz, 2019). We concluded that many online sports bettors would need to be invited to reach the targeted sample size of 300 online sports bettors for the case group. To enhance the probability of inviting possible GD cases, we used account holders flagged by the provider's AI algorithm as responsible gambling cases, to reach a sufficient proportion of players with GD. The provider uses an AI algorithm for responsible gambling purposes to classify risky gambling. The self‐learning algorithm does not screen for GD but looks amongst the player tracking data and the player communication data, for previously defined indicators that could predict ‘gambling related problems’ and evaluates them over time (Tipico Co. Ltd, 2023). Amongst others, indicators used are age, exposure, breadth and depth of involvement (LaPlante et al., 2014), complaint behaviour, or observation of strong emotions (in a Tipico shop, at the hotline, via e‐mail), and other indicators, that are not openly available. According to Tipico, the algorithm shows good diagnostic properties to indicate gambling related problems.

We asked the provider to send a standardised e‐mail to the account holders that we had preselected, which invites them to take part in the study in waves. After reaching about 300 AI‐flagged participants, we asked the provider to invite non‐AI‐flagged account holders. These had been randomly preselected by us to be sampled as controls. Different to the preregistration we did not match cases and controls according to age, gender, and age of betting account. The participation rate was similarly low across both groups (9.2%), so that a timely matching of cases and controls was organisationally not possible. Alternatively, we included (as preregistered) all three measures as control variables in all our analyses.

**FIGURE 2:**
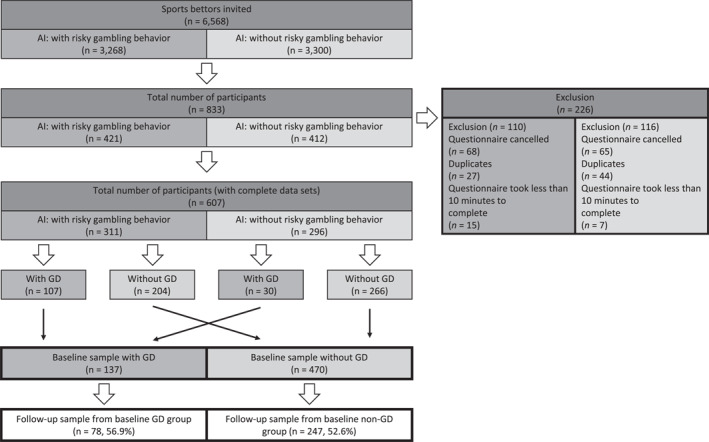
Flow chart of participants of the RIGAB study (Wirkus et al., 2023). AI = artificial intelligence, classification according to provider's algorithm. GD = gambling disorder assessed with DSM‐5 Stinchfield screening questionnaire. RIGAB, risky gambling behaviour.

For baseline recruitment, the sample had been divided into AI‐flagged and non‐AI‐flagged participants. To complete our data set, we connected the participants' online survey data to the player tracking data we had received from the provider before the initial online study had started (see Figure 2; Wirkus et al., 2023). Next, we analysed the results of the GD screening (DSM‐5; adapted from Stinchfield, 2002) and formed our actual case and control groups: with and without GD (see Figure 2).

### 2.3 Measures

The RIGAB study includes three main sources of data: (1) aggregated player tracking data, (2) two online surveys (baseline and 1‐year follow‐up), and (3) a standardised clinical interview and cognitive behavioural tasks from the in‐person assessment. An overview of the received player tracking data is given in Table 1, all other measures are depicted in Table 2 (Czernecka et al., 2023a). In the following, we describe the three sources of data in detail.

**TABLE 1:** Player account tracking data received from the provider Tipico.

Online sports player tracking data and account information	Description of monthly accumulated transaction data and of other account data obtained
Active days (monthly aggregate)	Sum of active days per month, that is, logged into the account and placed a bet
Bets (monthly aggregate)	Sum of bets per month, separate for prematch bets and live action bets
	Maximum number of bets on a single day within each month; separate for prematch bets and live action bets
Stakes (monthly aggregate)	Sum of stakes per month, separate for prematch bets and live action bets
	Variance of sum of stakes per month, separate for prematch and live action bets
Winnings (monthly aggregate)	Sum of winnings per month, separate for prematch and live action bets
Odds (monthly aggregate)	Average odds per month, separate for prematch and live action bets
	Variance of average odds per month, separate for prematch and live action bets
Deposits (monthly aggregate)	Amount deposited
	Variance of amount deposited
	Number of deposits
	Maximum number of deposits on a single day within each month
Withdrawals (monthly aggregate)	Amount withdrawn
	Variance of amount withdrawn
	Number of withdrawals
	Maximum number of withdrawals on a single day within each month
Age^a^	Age of accountholder (must provide ID to open account)
Registration date^a^	Date the account was opened
Last login	Date account holders last logged onto their account
Responsible gambling limits set by the account holder for	Frequency of deposit (set or not, frequency per month)
	Amount of deposits (set or not, amount per month)
	Frequency of stakes (set or not, frequency per month)
	Amount of stakes (set or not, amount per month)
	Frequency of losses (set or not, frequency per month)
	Amount of losses (set or not, amount per month)

^a^ Used as covariate in all analyses.

**TABLE 2:** Measures and instruments used in the RIGAB study (Czernecka et al., 2023a).

Variables and instruments	Baseline online survey	Follow‐up only survey	Nested design, in‐person assessment
Sociodemographic and gambling related information			
Sociodemographic data, incl. age,^a^ gender,^a^ education^a^	x	x	x
Debts	x	x	
Motives for gambling (based on the Gambling Motives Questionnaire (Braun, 2013; Stewart & Zack, 2008))	x	x	
Gambling participation during last 12 months (never—very often) regarding several types of gambling	x	x	
Preferred type of gambling	x	x	
Gambling frequency (days per week), only regarding online sports betting	x	x	
Gambling intensity (hours per gambling day), only regarding online sports betting	x	x	
Money spent on all types of gambling during last 12 months	x	x	
Gambling onset	x		
First form of gambling	x		
Gambling participation of social environment	x	x	
Changes of gambling behaviour during corona pandemic	x	x	
Perception of responsible gambling	x		x
Assessment of risk factors			
Alcohol consumption (QFI; Gmel & Rehm, 2004)	x	x	x
Tobacco consumption (QFI; Gmel & Rehm, 2004)	x	x	x
Impulsivity (short version of the German UPPS‐P; Wüllhorst et al., submitted) subscales: Sensation seeking, lack of premeditation, lack of perseverance, positive urgency, and negative urgency	x		
Difficulties in emotion identification (ubscale of TAS‐20; Kupfer et al., 2000)	x		
Difficulties in emotion regulation (ERQ; Abler & Kessler, 2009), subscales: Reappraisal, suppression	x		
Stress (PSS‐10; Schneider et al., 2020)	x		
Gambling disorder and comorbid disorders			
BSI‐18 (Spitzer et al., 2011)	x	x	
GSI global severity index, and subscales somatization, depression, anxiety			
Internal German translation of the DSM‐5 Stinchfield questionnaire (Stinchfield, 2002), adapted from Buchner et al. (2009): Gambling disorder screening	x	x	
BBGS (Hanewinkel et al., 2015)		x	
DIA‐X/M‐ CIDI (Wittchen & Pfister, 1997) assessing: Gambling disorder and selected comorbid mental disorders			x
Cognitive performance tasks on executive function and impulsive decision‐making			
Executive function (Wolff et al., 2021)			
Response inhibition (Go/No‐go task) IES			x
Updating/working memory (2‐back task) IES			x
Updating/working memory (number‐letter sequencing task) IES			x
Impulsive decision making (Pooseh et al., 2018)			
Delay discounting			x
Probability discounting (wins)			x
Probability discounting (losses)			x
Probability discounting (mixed gambles)			x

Abbreviations: BBGS, Brief Biosocial Gambling Screen; BSI, Brief Symptoms Inventory; ERQ, Emotion Regulation Questionnaire; GSI, global severity index; IES, inverse efficiency score; PSS, Perceived Stress Scale; QFI, quantity‐frequency‐index; RIGAB, risky gambling behaviour; TAS, Toronto Alexithymia Scale.

^a^ Used as covariate in all analyses.

#### 2.3.1 Player tracking data

We received monthly aggregated player tracking data for August 2020 until January 2021, which were originally intended to be the last 6 months preceding the baseline study. Due to organisational difficulties the start of the baseline study was postponed until May 2021. Tipico Co. Ltd. provided account data, and aggregated player tracking data (see Table 1), which are the base for all gambling behaviour variables. Tipico Co. Ltd. offers two kinds of online sports betting: live action bets and prematch bets. For prematch bets, bettors stake their bet before a sports game commences, for live action bets, bets can still be staked while a game is in progress. A previous study (Gray et al., 2012) has shown that people with risky gambling play more frequently in live action betting.

#### 2.3.2 Baseline and follow‐up online survey

##### GD measures

For the initial and the follow‐up online survey we used a screening for GD according to DSM‐5 criteria with an internal German translation of the dichotomous Stinchfield criteria (adapted from Buchner et al., 2009). The clinical DSM‐5 cut‐off (APA, 2013), to which we adhered in the study, is four criteria. Additionally, during the follow‐up online survey we used a brief 3‐item screening questionnaire for GD, the BBGS (Hanewinkel et al., 2015). The BBGS has been used more widely in Germany as it is very economical with high sensitivity and specificity. Hanewinkel et al. (2015) pointed out that the BBGS had not yet been validated in Germany. We have therefore added the BBGS to the follow‐up study to be able to validate its properties against the Stinchfield (adapted from Buchner et al., 2009) and a standardised clinical interview (Wittchen & Pfister, 1997). A highly valid and brief GD screening would be of interest for different types of prevention measures.

##### Putative risk factors

Basic research has found various putative risk factors for the development of GD. We assessed several risk factors in the initial online survey to validate them for sports betting. Selected risk factors were also assessed during the follow‐up online survey. In previous studies alcohol and tobacco use were reported to be elevated amongst players with GD (Chou & Afifi, 2011; Lorains et al., 2011; Sleczka et al., 2013). We assessed alcohol and tobacco use with a modified version of the quantity‐frequency‐index (QFI; Gmel & Rehm, 2004). For this modified QFI the average consumption per occasion is multiplied by the number of average weekly consumption opportunities. The QFI indicates the average consumption per week.

Impulsivity is one of the major personality traits known to correlate with GD (Chowdhury et al., 2017; Weinsztok et al., 2021). We have assessed impulsivity using a short version of the UPPS‐P Impulsive Behavior Scale (earlier version of Wüllhorst et al., submitted) including the subscales sensation seeking, lack of premeditation, lack of perseverance, positive urgency, and negative urgency. The subscales will be analysed separately.

People with GD also show altered emotion regulation (Jara‐Rizzo et al., 2019; Rogier et al., 2020). To verify these previous findings in our sample we have used the difficulties in emotion identification subscale of the Toronto Alexithymia Scale (TAS‐26; Kupfer et al., 2000). We further assessed altered emotion regulation using the Emotion Regulation Questionnaire (ERQ; Abler & Kessler, 2009). The ERQ has two subscales: reappraisal and suppression. The scores for these subscales are the means of the items of the respective subscale.

People with GD often show many comorbidities (Lorains et al., 2011; Sleczka et al., 2013). We have therefore included the Brief Symptom Inventory (BSI‐18; Spitzer et al., 2011), screening for somatization, depression, and anxiety. We will use these three subscales and the sum score of all items (global severity index, GSI) for analyses.

People with GD report heightened stress over a range of different self‐report measures in contrast to healthy controls (Elman et al., 2010), similarly greater problem gambling is associated with more perceived stressful live events (Ronzitti et al., 2018). In our study, participants were assessed with the Perceived Stress Scale (PSS‐10; Schneider et al., 2020). The sum score and subscales were included in analyses. Table 2 (Czernecka et al., 2023a) gives an overview of all self‐report measures for both online surveys.

##### Self‐reported gambling behaviour and gambling related information

In both online surveys, we have collected sociodemographic data as well as information on gambling behaviour. For instance, we asked about players' participation in different types of gambling, about the frequency and intensity of gambling participation, the motives for gambling (Gambling Motives Questionnaire; Braun, 2013; Stewart & Zack, 2008), gambling onset, and their net income. We also asked participants about their perception of the responsible gambling strategy of the provider. For all topics without references, we developed the questions ourselves making use of former surveys of our study group.

##### Covid‐related questions

The first Covid‐19 related lockdown in Germany started in March 2020, ending in May 2020. With the Covid‐19 pandemic still ongoing, the baseline online survey took place from May 2021 until August 2021. This could have some impact on our results. Previous studies have shown very heterogeneous results concerning the impact of the Covid‐19 pandemic restrictions on gambling behaviour ranging from an increase (Xuereb et al., 2021) to a decrease (Lischer et al., 2021) of gambling behaviour. During the planning of the study these studies had not yet been published. In order to consider a potential influence of the Covid‐19 pandemic, we also included questions that we developed ourselves asking about altered online gambling behaviour in relation to the pandemic in both, the baseline and follow‐up online survey. Analysing the data of the initial online study, we found that there was no change in online sports betting behaviour for two‐thirds of all participants (*n* = 405). From the other one‐third, 143 said there was a bit of a change (90 participants played a bit more, 53 played a bit less) and 59 participants said that there had been strong changes (31 played much more, 28 played much less). We therefore concluded that the Covid‐19 pandemic restrictions had some influence but not in a way that would not strongly bias our future results.

#### 2.3.3 In‐person assessment

##### Clinical interview for GD and comorbid disorders

Table 2 (Czernecka et al., 2023a) also gives an overview of the measures for the in‐person assessment. We conducted a clinical interview (DIA‐X/M‐CIDI; Wittchen & Pfister, 1997) to diagnose GD based on DSM‐5 criteria. Previous studies have shown that screening questionnaires ‐such as used in the online survey‐ may overestimate GD compared to clinical interviews (Kotter et al., 2019). We are overall interested in the explorative comparison of diagnostic properties of all our GD measures in a sample of German online gamblers. We also asked the participants about their perception of some aspects of responsible gambling strategies in general and selected aspects of the provider's strategy using questions we developed ourselves.

We also used the clinical interview (DIA‐X/M‐CIDI; Wittchen & Pfister, 1997) to assess comorbidity as a putative risk factor for GD. The DIA‐X/M‐CIDI was applied to assess lifetime and 12‐month prevalence of DSM‐IV‐TR affective disorders, somatic disorders, anxiety disorders, alcohol and tobacco use disorder as well as the respective age of onset for the examination of temporal relationships. The validity and reliability of mental disorders diagnosed with the DIA‐X/M‐CIDI has been demonstrated (Wittchen et al., 1998).

##### Cognitive behavioural tasks

We selected two computer task batteries to assess impaired executive function (EF) and impulsive decision‐making. The task batteries have already been successfully implemented in our lab (Kräplin et al., 2020, 2022). To implement these tasks, we used the Psychophysics Toolbox (Kleiner et al., 2007) in Matlab Runtime 2018a (The MathWorks Inc, 2018).

The EF task battery (Wolff et al., 2021) consists of a number‐letter sequencing task (switching), the 2‐back task (working memory), and a go/no‐go task (response inhibition). For all three tasks error rates and reaction times will be combined into inverse efficiency scores (IESs; Bruyer & Brysbaert, 2011) to account for individual differences in the balance of the speed‐accuracy trade‐off (Bogacz, 2015). Higher IESs implicate impaired EF. Previous studies report people with GD to show impaired EF (Kräplin & Goudriaan, 2018), which we plan to replicate.

The impulsive decision‐making battery (Pooseh et al., 2018) consists of (1) A delay discounting task, probability discounting tasks (2) for gains and (3) for losses, and (4) a mixed gamble task. For delay and probability discounting tasks, the discounting rate *k* of a hyperbolic value function (Mazur, 1987) will be used as an outcome. For the mixed gambles task, we will use a simple linear function in which loss aversion (*λ*) is the relative weighting of losses to gains in the participant's decision (Tom et al., 2007). Individuals with higher impulsive decision‐making are assumed to display higher *k* values in the delay discounting task, lower k values in both probability discounting tasks, and lower *λ* values in the mixed gambles task. In previous studies, people with GD have been shown to make more impulsive decisions (Kräplin & Goudriaan, 2018).

### 2.4 Data handling and data analysis

We will perform regression analyses and robust regression. In the sense of a sensitivity analysis, we will then compare the results, and should there be substantial differences, we will use the results of the robust method with less assumptions for our conclusions. As we will be performing significance tests on an individual hypothesis level correction for multiple tests is not necessary. Where necessary, we will use logistic regression.

As reported in our preregistration (https://osf.io/jbfhe), previous research has identified several sociodemographic factors as putative risk factors for GD and risky gambling behaviour; among them being young and male (Dowling et al., 2017; Hing et al., 2016) and education, as sports bettors are reported to have high levels of education (Hing et al., 2016). For all analyses, we will control for the influence of age, gender, highest level of completed education, and age of betting account as a proxy of gambling exposure. The rationale behind these covariates is comparable to previous studies in the field (Braverman & Shaffer, 2010; Gray et al., 2012; Hing et al., 2016). Additionally, in longitudinal analyses the number of days between two points of measurement, and where necessary baseline values, will be included as covariates.

## 3 RESULTS

As this is a study protocol, only the recruitment flow and the final sample size of the study parts are presented below. The initial online survey took place from May 2021 until August 2021. Six thousand five hundred and sixty‐eight account holders were invited, 833 participated, of those 226 were excluded because they did not complete the survey or were duplicates, that is, the personal code provided in the invitation email was used more than once. Eventually, the total number of participants with complete data sets amounted to 607 (311 participants AI‐flagged risky gamblers, 296 not). After analysing the GD screening, which was part of the baseline online survey, we formed our case and control groups. Cases (*n* = 137) fulfiled at least 4 DSM‐5 criteria (DSM‐5 cut‐off), controls (*n* = 470) <4 criteria. 555 participants gave consent and could be contacted to participate in the 1‐year follow‐up and the in‐person assessment (see Figure 2).

Data collection of the follow‐up online survey commenced 1 year after the initial online survey in May 2022, so that we reinvited all participants of the initial online survey exactly 364 days after filling in their baseline survey. Data collection was completed in August 2022. Of the 555 potential participants, 325 participated in the follow‐up (58.5%), 78 of the baseline GD‐group (56.9%) and 247 (52.6%) of the baseline non‐GD‐group.

The beginning of the in‐person assessment had to be postponed because of the Covid‐19 pandemic restrictions in winter 2021/2022. Due to organisational reasons, it solely took place with subjects from Berlin, Chemnitz, Dresden and Leipzig and concluded in May 2023 with 54 participants being interviewed and taking part in the experiments. All 54 took part in the initial online survey, 45 in both online surveys.

## 4 DISCUSSION

To the best of our knowledge the RIGAB study is the first comprehensive study on online gambling that combines three sources of data: (1) player tracking data, (2) self‐report questionnaires from two online surveys, including multiple GD screenings, and (3) in‐person clinical interviews and cognitive‐behavioural tasks. Combining these three fields, which are usually investigated separately, will allow for important theoretical as well as practical implications.

For theoretical etiological models, our study will provide new insights concerning the development of GD in online sports betting. The prospective nature of the study allows to explore predictive relationships between player tracking data, GD, and putative risk factors assessed by self‐report measures and cognitive‐behavioural performance tasks. To our knowledge, no other studies have yet investigated these relationships, be it particular to online gambling or to overall gambling. For example, the verification of the role of impaired emotion regulation in risky gambling behaviour and GD development will contribute significantly to etiological models of GD in sports betting. Investigating the predictive relationships is an important step towards causal conclusions, which are highly needed for the development of effective preventive measures and to individualise treatment.

A first practical implication is a much more holistic understanding of persons who participate in online sports betting. Because participants of the RIGAB study are from a naturalistic sample and not from a treatment sample, results can be transferred to other online gamblers, and in some cases probably to all gamblers.

We will also be able to better understand players' gambling motives, social background and environment, preferred types of gambling, gambling onset, and will be able to connect this information to player tracking data and to GD. Finally, we will find out about the players' perception of selected aspects of the provider's responsible gambling strategy and its overall use by the players. This so far unknown characterisation of individuals who participate in online sports betting is a further important step to better target prevention measures to the needs of this group.

As a second practical implication, the results of this study will allow us to develop and inform enhanced preventive and treatment measures for participants of online gambling based on the combined findings from previously rather distinct fields of gambling studies. For example, we could use the risk factors that are most prominent in online sports bettors as targets for early prevention measures to identify high‐risk individuals early and potentially intervene. More prominent risk factors could also be focused on in therapeutic interventions to treat patients with GD that participate in sports betting or online gambling.

## AUTHOR CONTRIBUTIONS

Theresa Wirkus conceptualized all preregistrations of the RIGAB study with Robert Czernecka, Anja Kräplin and Gerhard Bühringer as co‐authors. Robert Czernecka conceptualized and wrote the manuscript based on the preregistrations. Robert Czernecka and Anja Kräplin conceptualized the study including data and measures to be assessed. Theresa Wirkus conducted the study. All authors critically contributed to earlier drafts of the manuscript and approved the final version of the paper.

## CONFLICT OF INTEREST STATEMENT

Tipico provided the initial anonymised player tracking data and invited participants for the initial online survey, who were preselected by us. Per contract, Tipico had and has no influence on the design, data analyses, data interpretation and publication of the RIGAB study.

In the last 5 years RC was partly funded by an unrestricted research grant of members of the ‘Düsseldorfer Kreis’ (a group of key stakeholders from public and private gambling providers, research, and the support system) to TU Dresden and is now funded by an unrestricted research grant from the German Federal Ministry for Economic Affairs and Climate Action as part of the evaluation of the German Gambling Machine Regulation.

TW is funded by an unrestricted research grant provided by Tipico Co. Ltd. To TU Dresden.

GB received unrestricted grants for gambling research activities from various public and commercial gambling providers and regulatory agencies. He is a member of the ‘Düsseldorfer Kreis’ (a group of key stakeholders from public and private gambling providers, research, and the support system) and is also partly funded by an unrestricted research grant from the Federal Ministry of Economic Affairs and Climate Action as part of the evaluation of gambling hall regulations.

AK is mainly funded by the German Research Foundation (Deutsche Forschungsgemeinschaft—DFG) within the Collaborative Research Centre SFB 940 (project number 178833530) and additionally by an unrestricted research grant from the German Federal Ministry of Economic Affairs and Climate Action as part of the evaluation of the German Gambling Machine Regulation.

## Data Availability

As we received sensitive information, we will only be able to make the data available after we have finished the data collection to comply with data protection rights and ensure the participants' anonymity. Once data collection is completed, we will delete any personal information and make the anonymised data available on the Open Science Framework.
